# Temporary Anchorage Device: A Narrative Review

**DOI:** 10.7759/cureus.81617

**Published:** 2025-04-02

**Authors:** Khyati S Patel, Ishwa Parmar, Uday Kumar Digumarthi, Jaymin Parmar, Aafreen Quraishi, Kavya Patel, Alap Shah, Kinnari Shah, Bharvi Jani, Manvi A Arora

**Affiliations:** 1 Department of Orthodontics and Dentofacial Orthopedics, Karnavati School of Dentistry, Ahmedabad, IND; 2 Department of Orthodontics, Anil Neerukonda Institute of Dental Sciences, Visakhapatnam, IND; 3 Department of Dentistry, Karnavati School of Dentistry, Ahmedabad, IND; 4 Department of Orthodontics and Dentofacial Orthopedics, Karnavati University, Ahmedabad, IND

**Keywords:** anchorage, clinical application, orthodontics, site selection, temporary anchorage device, tooth movement, treatment planning

## Abstract

Anchorage control plays a crucial role in orthodontic treatment. When moving certain teeth into their desired position, it is essential to prevent any unintended movement of the teeth that are meant to remain in place. Temporary anchorage devices (TADs) are a significant advancement in modern orthodontics, providing a stable and reliable anchorage point for tooth movement. This review aims to discuss the types, clinical applications, benefits, and limitations of TADs, providing a comprehensive understanding of their role in contemporary orthodontic practice. The use of TADs enhances the precision and efficiency of orthodontic treatments, leading to faster results and improved patient comfort.

## Introduction and background

Orthodontic treatment aims to correct malocclusions, improve dental function, and enhance facial aesthetics through precise tooth movement. The success of orthodontic treatment depends on anchorage control. Anchorage, which refers to the resistance against unwanted tooth movement, is essential in orthodontic treatment for addressing both dental and skeletal malocclusions. In the past, orthodontists have relied on various anchorage methods, such as intraoral appliances, extraoral headgear, and intermaxillary elastics. These traditional methods have been widely used but often come with limitations, such as patient compliance issues, the potential for unwanted movement of other teeth, and less precise control over the desired movement. Temporary anchorage devices (TADs) have revolutionized modern orthodontics by providing a reliable and efficient means for controlling tooth movement. Temporary anchorage devices (TADs) are commonly utilized in orthodontics to facilitate complex tooth movements that are challenging to achieve with traditional appliances, such as anterior/posterior intrusion, the en masse distalization of upper/lower arches, molar uprighting, and molar distalization. The success of microimplant anchorage relies on the design of the microscrew, the correct insertion site, and precise surgical technique. With these factors in place, clinicians can achieve complete anchorage control without relying on patient compliance [1].

## Review

Materials and methods

The gathering of data for this narrative review was performed by conducting an electronic search for articles using the following databases: PubMed, Google Scholar, Science Direct, and Elton B. Stephens Company (EBSCO). The appropriate data were gathered using the search keywords temporary anchorage device, site selection, clinical application, anchorage, and tooth movement.

History

**Figure 1:**
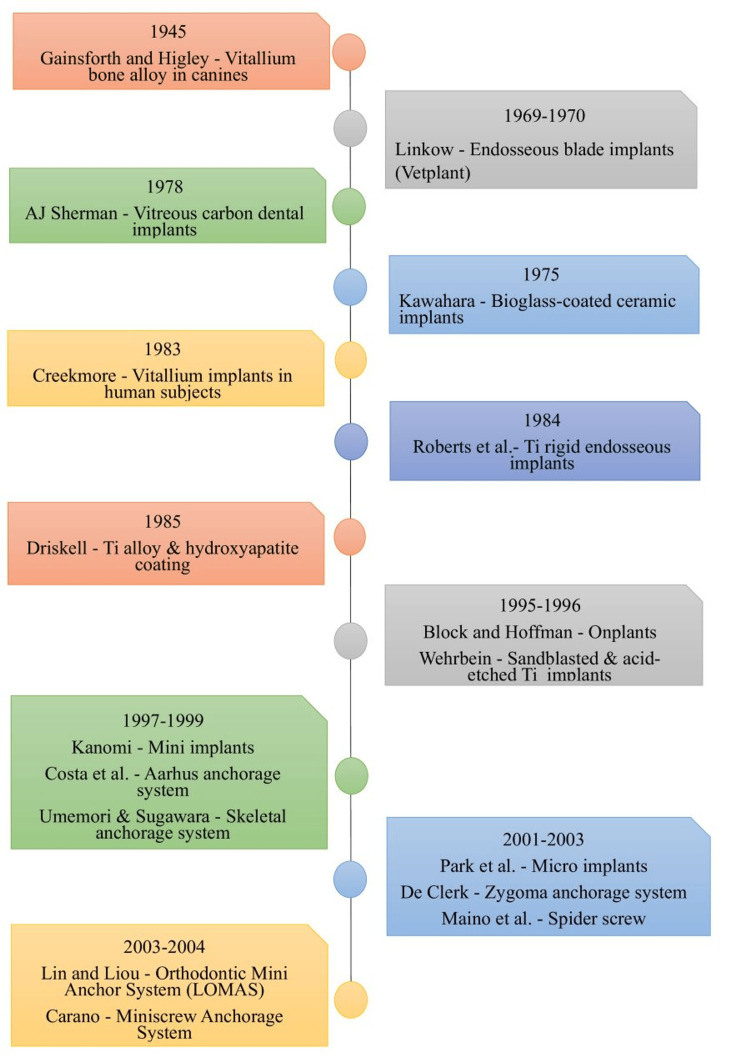
Figure depicting the evolution of implants in orthodontics with emphasis on key years and important milestones This figure was made using MS Word Document 2021 (Microsoft Corp., Redmond, WA) by Khyati S. Patel

Gainsforth and Higley (1945) first published the use of subperiosteal Vitallium implant to retract maxillary canines in dogs [2]. In 1969, Linkow [3] described endosseous blade implants with perforation for orthodontic anchorage [4]. In 1983, Creekmore and Eklund reported the possibility of skeletal anchorage in orthodontics [5]. Roberts et al. (1990) used a traditional two-stage endosseous implant in the retromolar area to effectively reinforce anchorage while successfully closing the extraction site of the first molar in the mandible [6]. In 1994, Roberts et al. used an anchorage implant in the retromolar region about 5 mm distal to the mandibular third molar for the first half of space closure [7]. Turley et al. (1988) used endosseous implants in dogs as anchorage for the application of a variety of orthodontic and orthopedic forces [8]. In 1995, Block and Hoffman introduced the concept of using the palate as a location for anchorage devices with the creation of the onplant [9]. In 1997, Kanomi first reported the clinical use of mini-implants for orthodontic anchorage [10]. In 2002, Chung et al. introduced the C microimplant system [11]. Maino et al. (2003) introduced a spider screw system implant for skeletal anchorage [12]. Kyung et al. introduced another microimplant system called the "Absoanchor" (Figure 1) [13].

Classification

There are eight types of classification based on different location, shape and size, composition, bone integration, use, configuration design, form, and surface structure [14-18]. The details about the classification are given in Table 1.

**Table 1:** Classification of temporary anchorage device This table was made using MS Word Document by Khyati S. Patel

Classification details				
Based on location	Subperiosteal	Transosseous	Endosseous	-
Based on shape and size	Conical (cylindrical)	Miniplate implants	Disimplants (onplants)	-
Based on composition	Stainless steel	Cobalt-chromium-molybdenum	Titanium and ceramic implants	Miscellaneous
Based on bone integration	Osseointegrated	Nonosseointegrated	-	-
Based on use	Orthodontic implants	Prosthodontic implants	-	-
Based on configuration design	Root form implants	Blade/plate implants	-	-
Based on form	Solid	Hollow	Vented	-
Based on surface structure	Threaded or nonthreaded	Porous or nonporous	-	-

Types of anchorage

Miniscrew implants can offer two types of anchorage. Direct anchorage involves using an anchorage device that is firmly attached to the bone, establishing a stable and fixed reference point for managing tooth movement. Indirect anchorage, on the other hand, is a technique in which the anchorage device is not directly connected to the teeth that require movement but instead relies on other teeth or structures in the mouth for support [19].

Parts of orthodontic implants

**Figure 2:**
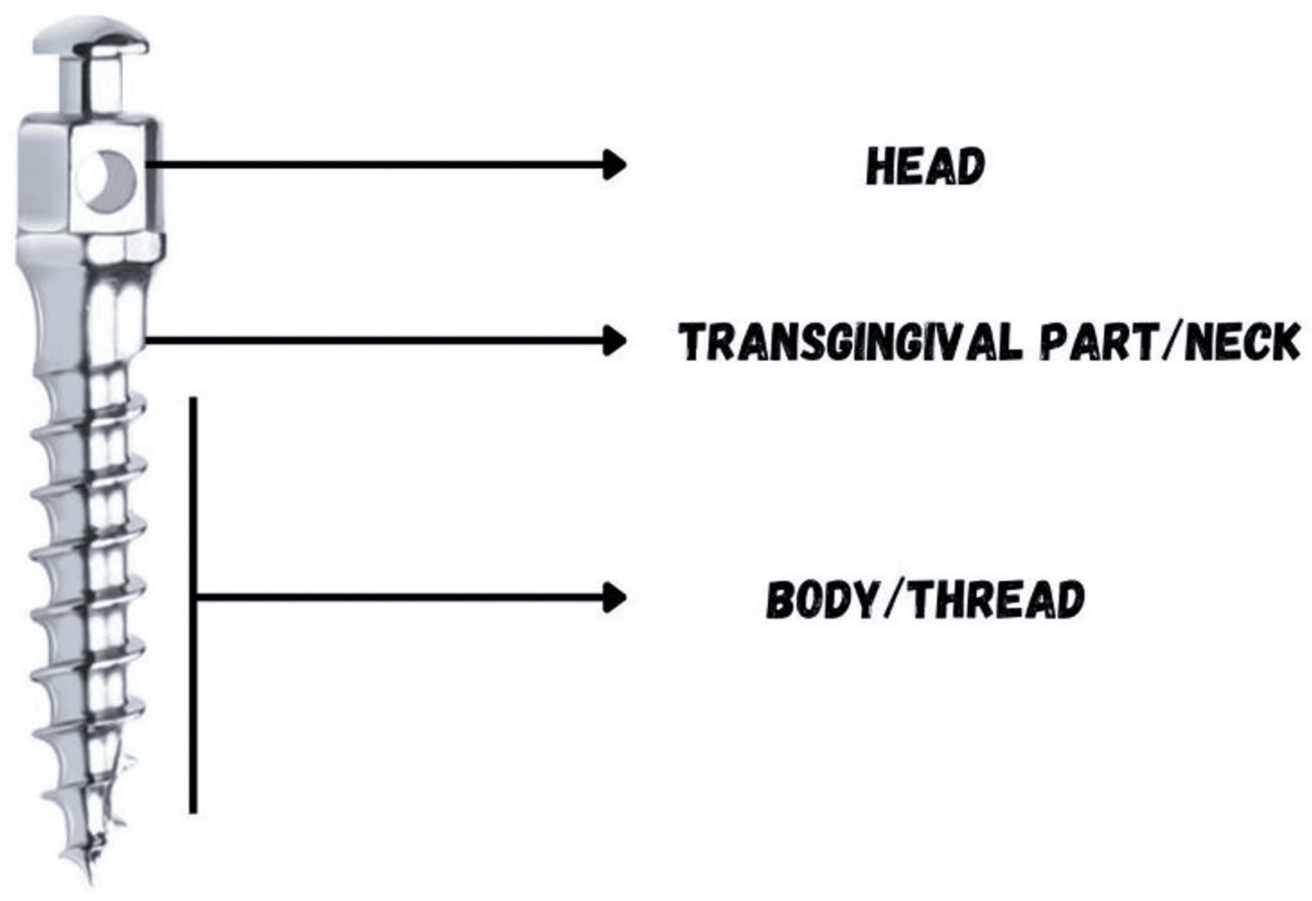
Parts of the temporary anchorage device This figure was made using Canva (Canva Pty Ltd, Sydney, Australia) by Khyati S. Patel (https://www.canva.com/design/DAGiR50-6pU/SUPnNMgHhjA82k6OX9ZBtQ/edit?utm_content=DAGiR50-6pU&utm_campaign=designshare&utm_medium=link2&utm_source=sharebutton)

The temporary anchorage device consists of three components: the head, neck, and body (screw) (Figure 2). First, the head is the top part of the implant where attachments or orthodontic appliances are connected. Then, the neck is the part that is present between the platform and head, which serves as the attachment site for various accessories such as an elastic NiTi coil spring or other orthodontic attachments. The body is the main portion of the implant, often threaded, which is inserted into the bone to provide stable anchorage [20,21].

Head type classifications

There are different head types, which are characterized as small head, long head, circle head, fixation head, bracket head, and hook head. First, a small head is ideal for situations with limited spaces, as its compact size allows for easier placement. Second, a long head provides a larger surface area for force distribution, which helps improve stability and ensures more efficient and controlled tooth movement during orthodontic treatment. Third, a circle head offers versatility by allowing forces to be applied from various angles and providing greater flexibility in treatment and more control over tooth movement. Fourth, the fixation head helps secure auxiliary components and provides support for complex orthodontic treatments, ensuring stability and precise control during movements. Fifth, the bracket head enables the direct attachment of archwires, allowing for efficient force application and precise tooth movement. Lastly, the hook head offers attachment points for elastics or other auxiliary elements [20].

Miniscrew length and diameter

The size ranges are as follows: the length varies from 4 mm to 12 mm, while the diameter ranges from 1.2 mm to 2.7 mm. The commonly used miniscrew thickness is 1.5 mm. However, a thickness of 1.2 mm is used between the lower incisors due to the limited inter-radicular space [22-24]. In a study, larger-diameter (2.5 mm) monocortical screws provide increased anchorage force resistance compared with smaller-diameter (1.5 mm) monocortical screws in both the mandible and the maxilla. Smaller-diameter (1.5 mm) bicortical screws provide anchorage force resistance at least equal to larger-diameter (2.5 mm) monocortical screws [25].

Site consideration in the placement of TADs

Anatomical Structures at the Site of Placement

When placing TADs, there is a risk of perforating the roots of the adjacent teeth, nerves, blood vessels, bone, and sinuses in the area around the intended placement site. Careful planning and precision are crucial to avoid damaging these vulnerable structures [26].

Soft Tissue Characteristics

The quality of the soft tissue plays a crucial role in determining the success of anchorage provided by TADs. Ideally, TADs should be placed in attached gingiva as it is resistant to inflammation. The firm and attached gingiva is preferred over movable mucosa for better stability and tissue response [26].

Patient Comfort

Patients typically experience little to no pain after the routine placement of TADs. The procedure generally causes little to no discomfort, and if any pain does occur, it typically lasts no longer than a day or two [26].

Bone Characteristics

**Figure 3:**
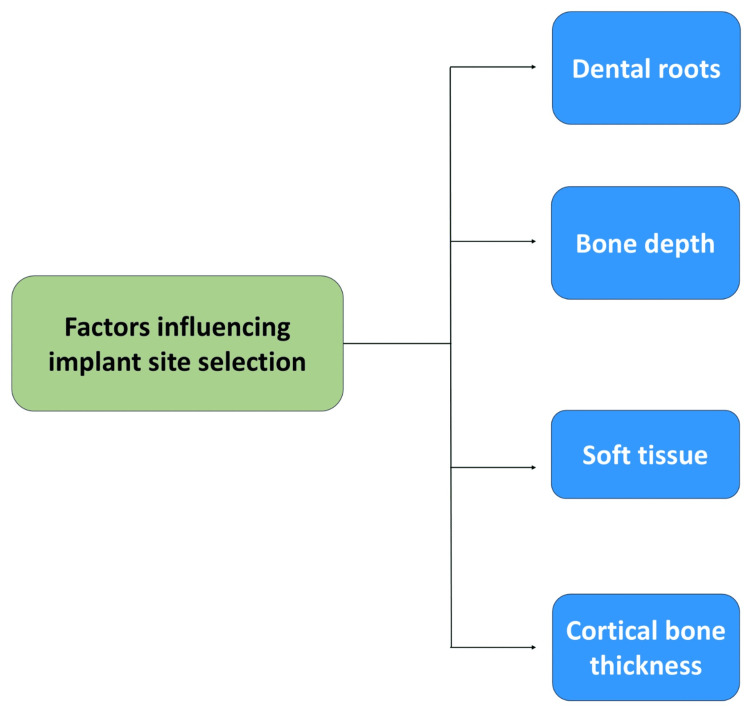
Factors influencing implant site selection This figure was made using MS PowerPoint Presentation (Microsoft Corp., Redmond, WA) by Khyati S. Patel

The integrity of temporary anchorage devices (TADs) is significantly influenced by the condition and amount of cortical bone. Bone thickness and density can vary not only between different anatomical sites in the same patient but also across different patients. The porous bone (D3 or D4) is present in the maxillary alveolar bone, and the denser bone (D2 and D3) is present in the mandibular alveolar bone (the Misch classification). The anterior regions generally have denser bones than the posterior areas. The maxillary cortical bone is thicker in the palate compared to the buccal surface. Specifically, the palatal cortical bone is consistently uniform, with a thickness of 4 mm or more apical to the cementoenamel junction (CEJ). Conversely, the cortical thickness of the mandibular buccal alveolar bone increases as it extends toward the ramus. The midpalatal region has a good-quality cortical bone with sufficient volume for TAD placement, and the retromolar pad area in the mandible also consists of dense cortical bone [9,10,27]. Factors influencing the precise placement of an orthodontic mini-implant are shown in Figure 3.

Inter-radicular sites for TAD placement

The appropriate sites for TAD insertion lie between the clinically invisible crestal bone margin and the clinically visible mucogingival border [28]. At least 3 mm of space between roots is generally considered necessary for the safe placement of TAD. A safety depth of at least 4 mm is required in the buccolingual direction to ensure sufficient space, as most monocortical TADs do not penetrate the opposite cortical bone [29]. The narrowest inter-radicular space should be greater than 3 mm. The thickness of the bone over the narrowest inter-radicular region must be adequate to support the length of the TAD [29]. Poggio et al. provided an anatomical map to assist in the safe placement of TADs between roots of the teeth [30].

Most commonly used sites in the maxilla

The most commonly used sites in the maxilla are as follows: the infrazygomatic region, zygomatic buttress; between the second premolar and the first permanent molar; between the first and second permanent molar buccally; between central incisors; the maxillary tuberosity region; palatal areas; and the midpalatal region [30,31].

Most common sites in the mandible

The most common sites in the mandible are as follows: between central incisors, between mandibular canine and premolar buccally, between the second premolar and the first permanent molar, between two permanent molars, the retromolar region, and the mandibular symphysis [30,31].

Extraradicular sites

Palatal Site for TAD Insertion

**Figure 4:**
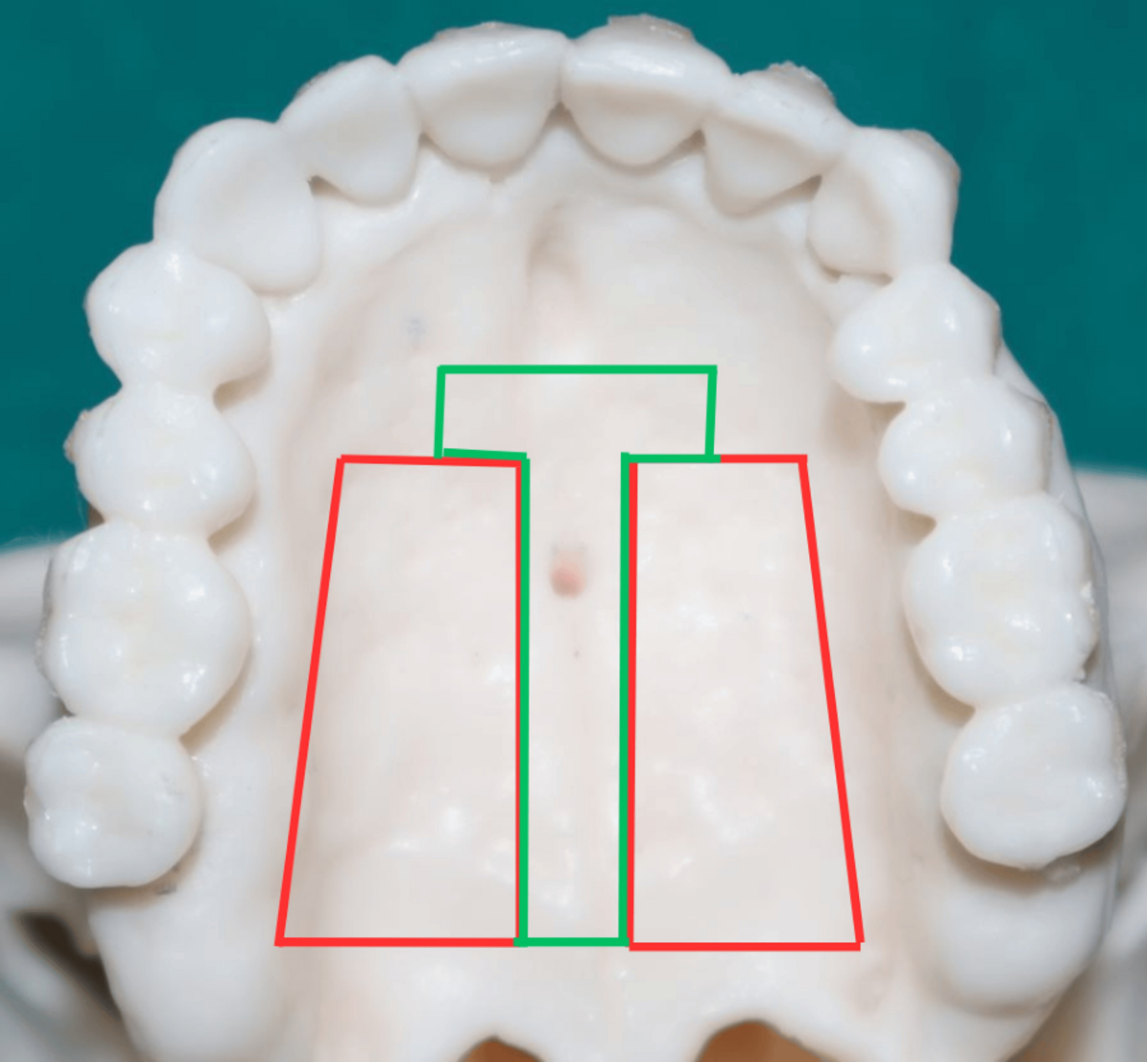
Recommended insertion site posterior to the palatal rugae ("T-Zone") The photograph was taken by Khyati S. Patel and edited using Canva (https://www.canva.com/design/DAGiVkgcEdI/mqSaTUC6evumQoUI7G0WIQ/edit?utm_content=DAGiVkgcEdI&utm_campaign=designshare&utm_medium=link2&utm_source=sharebutton)

The anterior palate is an ideal site for TAD placement due to its high bone quality, thin soft tissue, and minimal risk of root damage or interference with the teeth. It enables the incorporation of larger TADs with increased stability [32,33]. The midsagittal and paramedian regions of the hard palate are good options for TAD placement, as they are easily accessible and covered by attached mucosa, providing excellent peri-implant conditions [34-36]. In the lateral and posterior areas of the palate, bone volume is reduced, limiting TAD placement to the median insertion in the posterior palate [37,38]. Proximity to structures such as incisor roots and incisive canals should be considered. Insertion directly into the palatal rugae can be difficult. Instead, the area just posterior to the palatal rugae, known as the "T-Zone," offers a more suitable location due to sufficient bone volume. As a general guideline, TADs should be placed posterior to the third palatal rugae within the T-Zone. Typically, at 3-4 mm distal to the incisive foramen and 3 mm around the palatal suture, the thickest vertical bone is present. Studies have shown a mean bone thickness of only 2.94 mm at the palatal suture. Therefore, TADs should be positioned 3-6 mm paramedian to the suture and 6-9 mm distal to the incisive foramen for optimal placement (Figure 4) [36,39].

*Infrazygomatic Crest (IZC) *Region* for TAD Placement*

**Figure 5:**
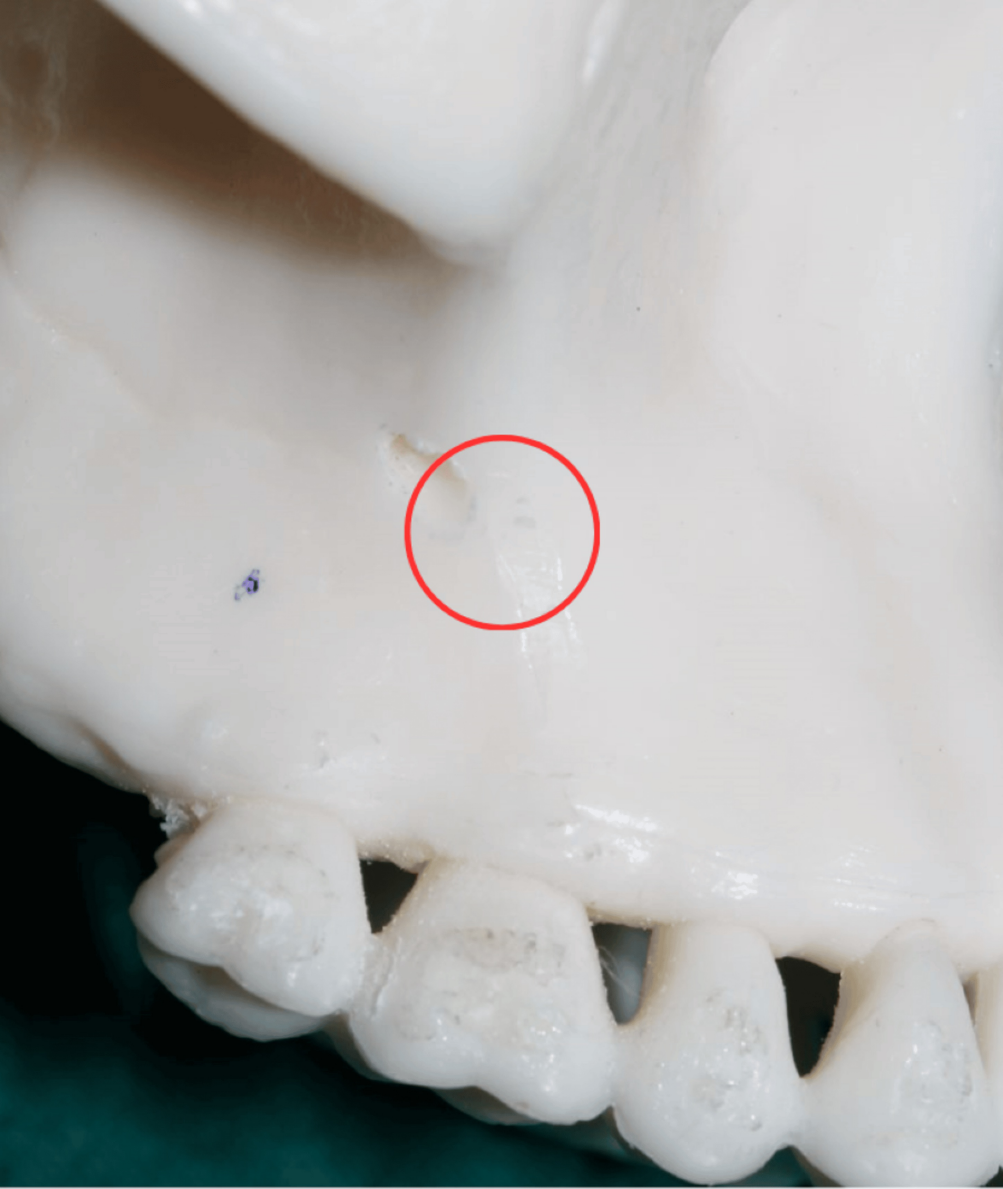
Location of the infrazygomatic crest The photograph was taken by Khyati S. Patel and edited using Canva (https://www.canva.com/design/DAGiV4rIYt0/GVuxBjiQ49eDNaiZhPgWyA/edit?utm_content=DAGiV4rIYt0&utm_campaign=designshare&utm_medium=link2&utm_source=sharebutton)

The IZC region is a cortical bone pillar at the zygomatic process of the maxilla. It runs along a palpable bony ridge between the alveolar and zygomatic processes of the maxilla. The TAD placement site is typically located between the second premolar and first molar in younger patients, while in adults, it is positioned above the first molar. The ridge extends over 2 cm superiorly to the zygomatic-maxillary suture. TADs should be placed with approximately 1.5 mm of clearance from the soft tissue to the screw platform to avoid soft tissue irritation. The attached gingiva near the first molar is about 1 mm thick, with 1.1-1.3 mm cortical bone thickness. For soft tissue clearance and cortical bone engagement, IZC TADs generally penetrate the bone or sinus by 3.5-7.5 mm, with an 8 mm screw typically providing adequate stability. For optimal buccal bone engagement, TADs should be oriented at a 55°-70° angle inferior to the horizontal plane. The alveolar bone on the buccal surface of the second molar is preferred because it is a thicker bone. The IZC TAD sites are typically inferior to the anatomical zygomatic crest for clinical convenience, allowing for more stable placement and avoiding soft tissue interference (Figure 5) [40-42].

Maxillary Tuberosity for TAD Placement

**Figure 6:**
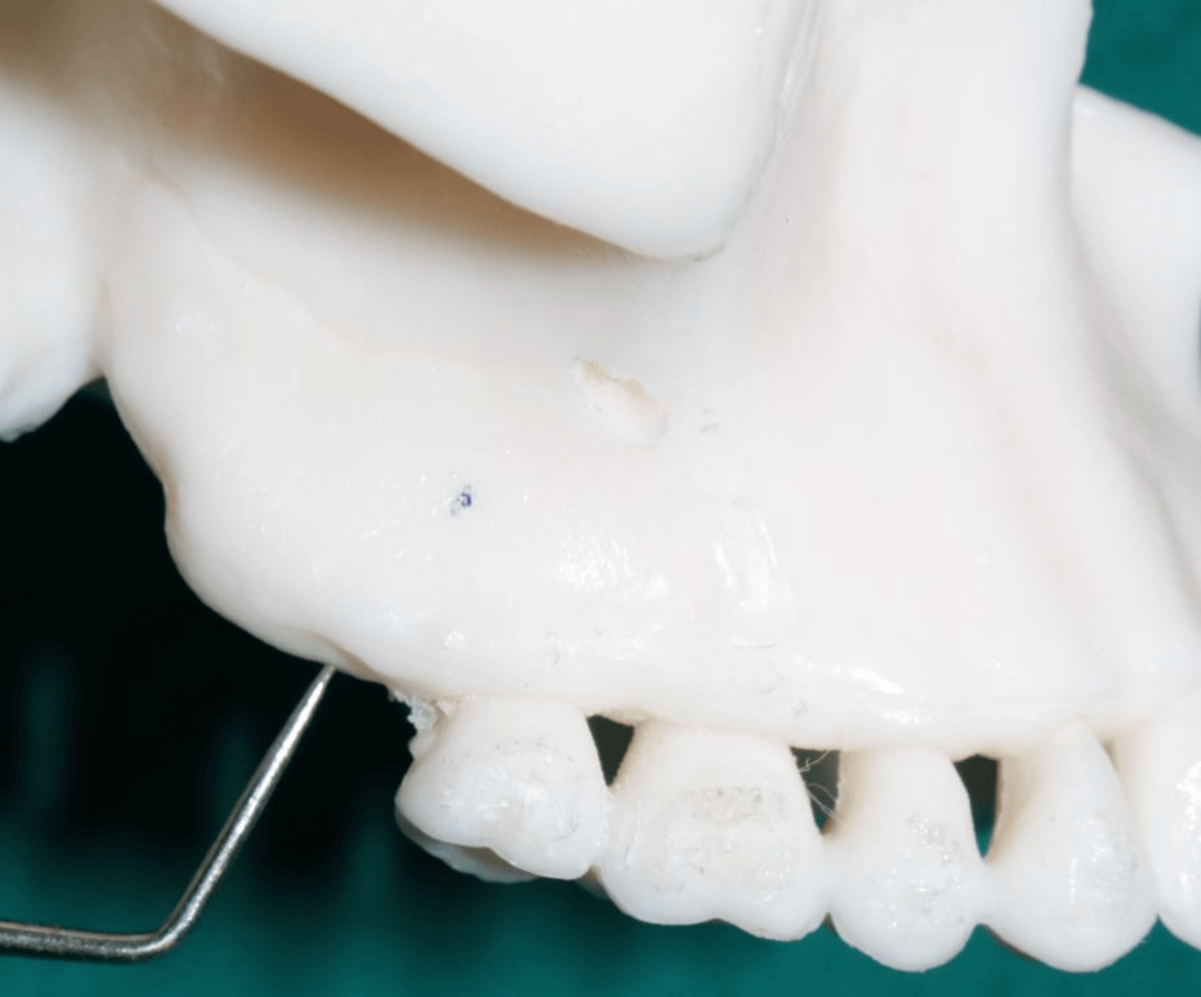
Orientation of the temporary anchorage device in the maxillary tuberosity region The photograph was taken by Khyati S. Patel and edited using Canva

The maxillary tuberosity is located at the lower part of the infratemporal surface of the maxilla. It is a rounded prominence, especially after the eruption of the wisdom tooth. The lateral side of the tuberosity is rough, articulating with the pyramidal process of the palatine bone and sometimes with the lateral pterygoid plate of the sphenoid. The posterior maxillary region typically consists of bone type III or IV, which is characterized by thin cortical bone and low-density trabecular bone. Bone height is often insufficient for implant placement due to the maxillary sinus. This makes achieving high primary stability more challenging. Although the bone quality is relatively poor (Misch D3 or D4 categories) at the maxillary tuberosity, the soft tissue is thin in this area, allowing the use of 6-7 mm-long TADs. A mini-implant with a diameter of 1.3-1.5 mm is recommended for areas with cancellous bone and low density, such as the maxillary tuberosity. TADs should be placed at an angulation of 20°-40° relative to the occlusal plane in a vertically directed manner (Figure 6) [43,44].

Mandibular Buccal Shelf (MBS) Area

**Figure 7:**
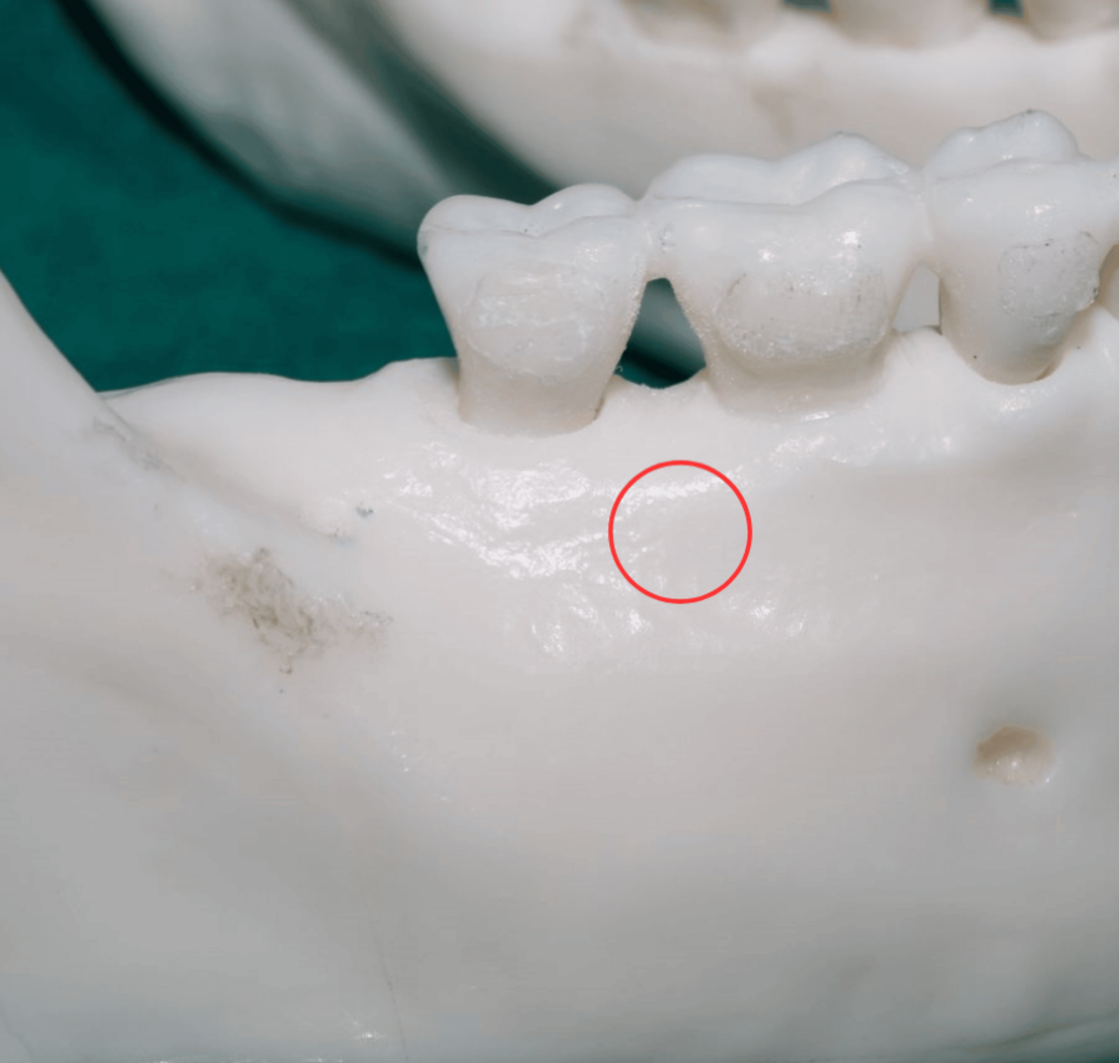
The location of the buccal shelf screw placement The photograph was taken by Khyati S. Patel and edited using Canva (https://www.canva.com/design/DAGiWL_A7j4/VTWvGeO90wvvVmQ1TVXEHQ/edit?utm_content=DAGiWL_A7j4&utm_campaign=designshare&utm_medium=link2&utm_source=sharebutton)

The MBS is situated bilaterally in the posterior portion of the mandibular body, located buccal to the roots of the first and second molars, and anterior to the oblique line of the mandibular ramus. The buccal bone, lateral to the distal root of the second molar, approximately 4 mm buccal to the cementoenamel junction (CEJ), is an optimal site for insertion. For specific biomechanical needs, insertion may also be considered lateral to the mesial root of the second molar, though it may require a more apical placement to achieve sufficient buccal bone thickness. Assessing the thickness of the cortical bone before insertion is essential. Predrilling may be required to enhance primary stability and minimize the risk of excessive torque and screw failure. The optimal angulation of the MBS for TAD insertion is approximately 38°, which should match the angulation of the TAD to align with the axial inclination of the molar (Figure 7) [45,46].

Ramus Area for TAD Placement

To address the limitations of traditional TADs (such as retromolar and inter-radicular) in managing horizontal impactions, Chang et al. developed a 2 mm-diameter stainless steel (SS) bone screw, suitable for dense cortical bone sites such as the mandibular buccal shelf (MBS) [46]. The MBS screw is placed lateral to the first and second molars, avoiding interference with horizontal impactions or the path of tooth movement within the alveolar process. The ramus of the mandible is quadrilateral in shape, with two surfaces, four borders, and two processes. The ideal site for TAD placement in the ramus is midway between the external and internal oblique ridges of the ascending ramus, approximately 5-8 mm above the occlusal plane, providing a direct line of traction without occlusal interference [47].

Retromolar Pad for TAD Placement

TADs are positioned in the area of the retromolar pad when a complete distal retraction of the mandibular dentition is intended. From an anatomical perspective, the retromolar pad is a triangular region defined medially by the temporal crest, laterally by the anterior border of the ramus, and posteriorly by the area near the third molar [48]. Also referred to as the piriformis papilla, the retromolar pad is a compressible elevation in the mucosa situated over the retromolar triangle, measuring approximately 11.2 mm in length and having a maximum transverse diameter of 7.9 mm. The best location for temporary anchorage devices (TADs) is slightly to the buccal side of the buccolingual midpoint of the retromolar triangle, creating a bull's-eye configuration. It is crucial to refrain from placing the TAD on the lingual side of the internal oblique ridge as this area contains a bony undercut and is situated near the lingual nerve and blood vessels. To identify the best spot for TAD placement, palpating the outer oblique ridge can assist the clinician in finding the ideal location [49].

Symphyseal Area for TAD Placement

**Figure 8:**
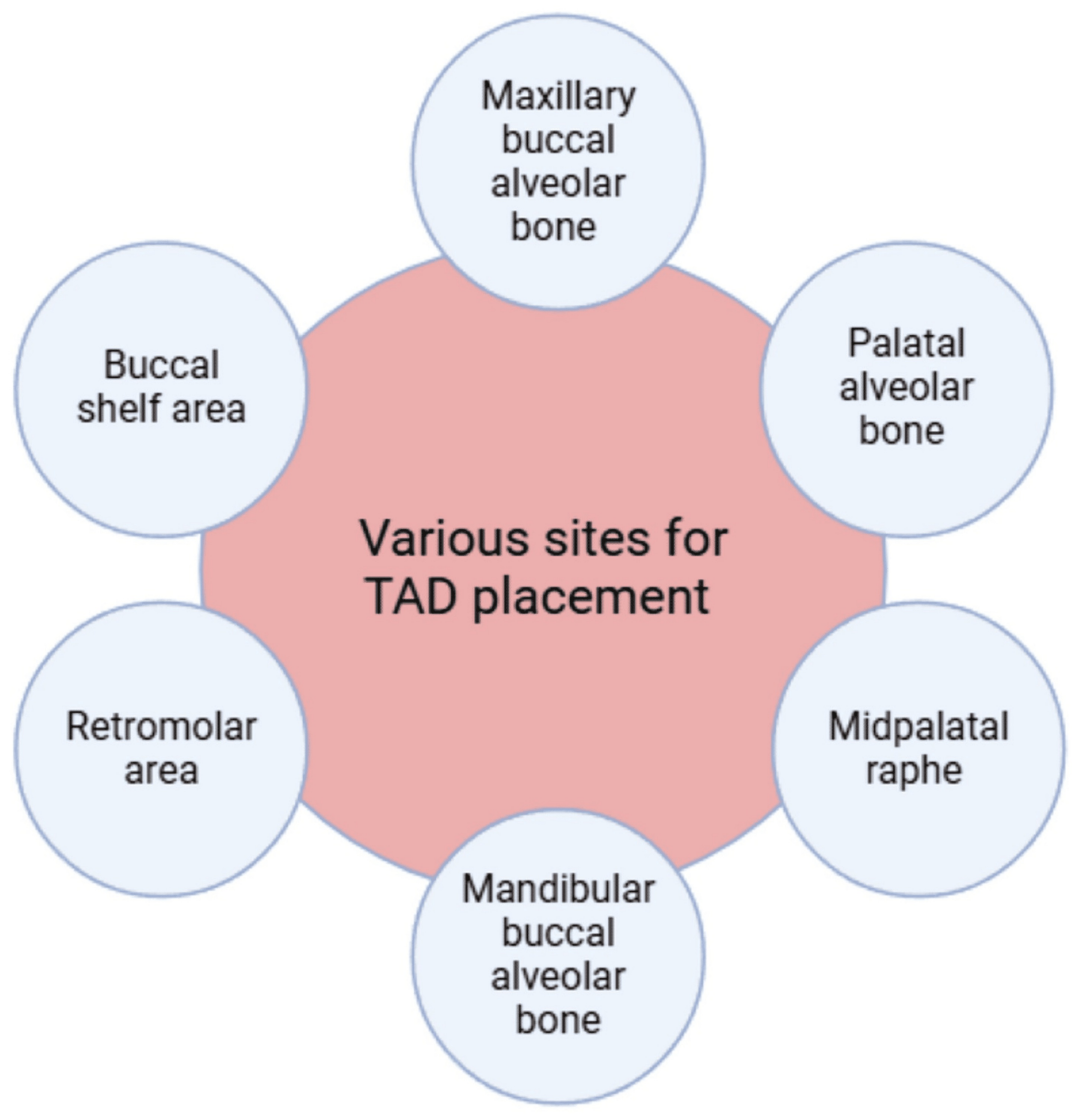
Sites for TAD placement This figure was made using BioRender (BioRender, Toronto, Canada) by Khyati S. Patel (https://BioRender.com) TAD: temporary anchorage device

TADs are placed in the symphysis area to secure fixed functional appliances in developing patients with skeletal Class II malocclusion, and they are also utilized for Class III elastics and the intrusion of mandibular incisors in individuals with a deep bite. The symphyseal area is an optimal site for screw placement because it has a thick cortical bone and is easily accessible. The upper outer surface of the symphysis may exhibit a subtle median ridge, indicating where the two halves of the fetal bone fused. Inferiorly, this ridge branches out to create a triangular mental protuberance, with the base being depressed and the sides elevated as mental tubercles. These characteristics form the chin. The mental foramen, through which the mental nerve and blood vessels pass, is situated beneath the space between the premolar teeth, usually near the second premolar [50]. Various sites for TAD placement are shown in Figure 8.

Indication

Temporary anchorage devices (TADs) are versatile tool in orthodontics, especially when traditional anchorage systems are insufficient. They can be utilized in numerous clinical scenarios, such as delivering absolute anchorage during maximum retraction, ideal for noncompliant patients. TADs prove particularly advantageous in adult orthodontic treatments, where they aid in complex tooth movements that may not be feasible with standard approaches.

They are effective in situations where the first molars are absent and can also assist in correcting deep bites, closing spaces left by extractions, and aligning tilted occlusal planes. TADs play an important role in correcting midline issues and addressing midline asymmetry or occlusal inclination. Moreover, they can assist in the alignment of impacted canines, as well as in the upright positioning and extrusion of impacted molars. TADs are beneficial for molar intrusion and support the distalization of maxillary molars and mandibular teeth. Additionally, they can be useful in managing vertical skeletal discrepancies, offering a broad array of solutions for difficult orthodontic cases [51,52].

Contraindications

Although there are no definitive contraindications for the insertion of orthodontic mini-implants, they are typically not advised for individuals with mental health disorders, significant systemic issues such as osteoporosis, blood-related conditions, or a background of substance abuse, including alcohol. Furthermore, candidates with inadequate bone quality or diabetes might not be the best fits for implant placement [51,52].

Insertion method

There are two types of miniscrew implants. Self-drilling miniscrews have uniquely engineered tips and cutting flutes that operate similarly to a corkscrew, enabling them to be directly inserted into the bone without requiring any predrilling. Non-self-drilling miniscrews require a pilot hole to be drilled before insertion. While this gives more control in denser bone, it adds complexity to the procedure and carries the risk of damaging nearby structures, such as nerves or tooth roots, if not done carefully [53-55]. An acrylic template or surgical guide is typically used to ensure the precise placement of the miniscrew and minimize the chances of error. The procedure is usually carried out under local anesthesia or infiltration, ensuring the patient remains comfortable throughout the process. The soft tissue at the planned insertion site is removed using a soft tissue punch, allowing for clear access to the bone. A drill bit that spins at a maximum of 1000 revolutions per minute (rpm) is used to create a pilot hole. The hole should be 2-3 mm deep and 0.3 mm smaller than the diameter of the miniscrew. The miniscrew is then placed using a specialized screwdriver, ensuring it is positioned at the correct angle and depth for stability. The choice between self-drilling and non-self-drilling screws largely depends on the bone's thickness and density [55,56].

Stability of implants

The stability of implants is a major concern, whether the implant relies on osseointegration or mechanical retention. It consists of two key types of stability. Primary stability, or initial stability, is achieved right after the insertion of an implant. It is a critical factor for the healing process and for determining when the implant can safely be loaded with functional forces. Secondary stability refers to the increased stability that occurs after implant placement as a result of bone regeneration and remodeling. Over time, the bone integrates with the implant, enhancing its stability and securing it more firmly in place [1,16].

Factors affecting success rate

The effectiveness of temporary anchorage devices (TADs) ranges from 80% to 100%, depending on various factors related to the device, patient, dentist, and implant location. Device-related factors include the risk of miniscrew fracture, which can occur due to a thin diameter or low strength; using a solid, conical screw design can help prevent this. Infection is another common cause of failure, and selecting screws with smooth transmucosal parts can reduce infection risks. Dentist-related factors include applying excessive pressure during insertion, which can fracture the cutting tip of self-drilling screws, and over-tightening the screw, which can lead to loosening. It is also important to apply stable, consistent forces during insertion to avoid loosening. Patient-related factors include the thickness of the cortical bone; if it is less than 0.5 mm, primary stability may not be achievable, and an alternative site may be necessary. Additionally, thick mucosa increases the distance between the point of force application and the screw's center of resistance (CRes), requiring the use of longer screws for proper stability. Addressing these factors during planning and placement can significantly improve the success rate of TADs [57-60].

Implant failures

Immediate Failures

Immediate failures occur right after the insertion of the miniscrew. Immediate failures in miniscrew placement can commonly result from choosing an incorrect insertion site, which leads to instability. Improper handling during the insertion process, such as wobbling the screw or altering the angle abruptly, may also result in failure. Placing the screw too soon after a tooth extraction is another contributing factor, as the recent extraction site may be unstable and affect the success of the screw. Thick overlying soft tissue or mucosa, including dense gum tissue, can hinder proper placement and compromise the screw's stability. Finally, the excessive tightening of the screw can cause it to fracture when it reaches the periosteum, the protective membrane around the bone, leading to failure [60].

Delayed Failures

Delayed failures can occur during orthodontic treatment, even if the initial placement was successful. These include excessive loading from elastic components, where too much force from elastic or other orthodontic devices can cause the screw to loosen. A sudden impact on the miniscrew head during chewing can also lead to failure, as biting or chewing places sudden pressure on the screw. Another issue is contact with the tooth root; if the screw comes into contact with the root surface, it can cause loosening or failure. Furthermore, excessive or insufficient bone remodeling can affect the screw's stability if the bone does not remodel properly around it. If a miniscrew fails, reinserting it into the same site often leads to repeated failure. In such cases, it is recommended to place the screw at an adjacent site. If the original location is essential, using a miniscrew with a wider diameter after 2-3 months can increase the chances of successful reinsertion [60].

Loading and anchorage consideration

Orthodontic miniscrews are subjected to loading immediately after placement; however, they are exposed to minimal forces only to ensure appropriate integration while reducing the likelihood of complications [23,61]. Liou et al. recommended ensuring a minimum of 2 mm distance between the implant and the roots of the adjacent teeth since implants can move as a result of orthodontic pressure [62]. Research has determined that mini-implants provide a reliable form of anchorage; however, they do not remain entirely immobile during the orthodontic loading phase. In certain patients, mini-implants may move in reaction to the applied orthodontic forces [63]. A study was carried out to assess the stability of miniscrew implants, taking into account the timing, amount, and site of force application. The findings endorse the notion that applying light forces (25-50 g) to miniscrew implants immediately can result in high success rates, facilitating meaningful and effective tooth movements. These results were unaffected by the magnitude of force or the site of its application [64].

Implant removal

The implant can be unscrewed using a screwdriver, with or without the application of topical or local anesthesia. If the implant cannot be removed, it is recommended to wait for 3-7 days, as the microfractures caused during the procedure may lead to the screw loosening over time [19].

Clinical applications

**Figure 9:**
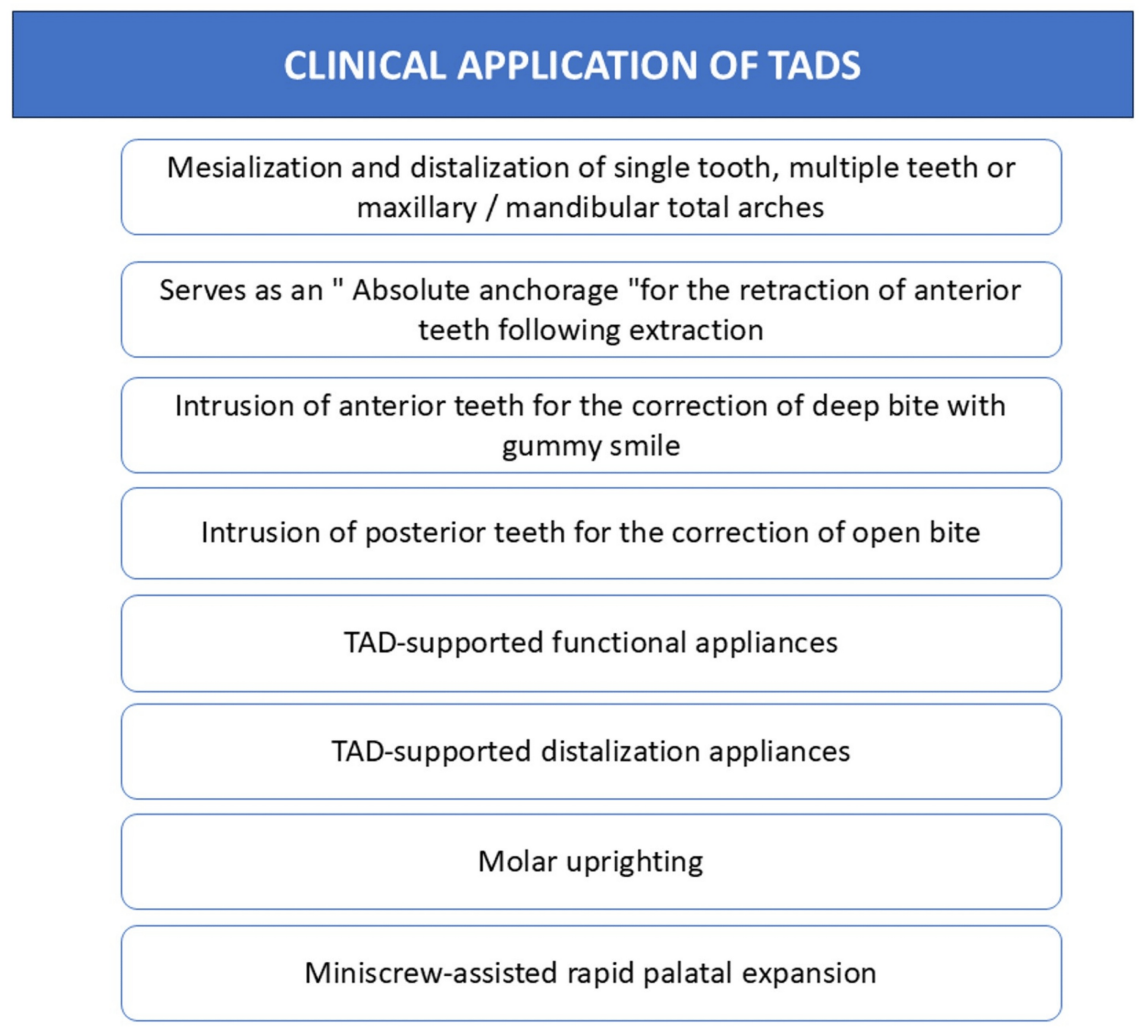
Clinical application of temporary anchorage device (TAD) This figure was made using MS PowerPoint Presentation by Khyati S. Patel

The mini-implant facilitated notable intrusion and alignment of the maxillary incisors, without depending on patient cooperation. This anchorage method was highly effective in significantly improving the patient's dental deep bite and gummy smile [65,66]. Miniscrews offer a reliable and minimally invasive solution for maxillary molar intrusion. For the intrusion of mandibular molars, miniplates are the favored option [67,68]. Miniscrew-supported distalizers are commonly placed in the anterior palate to treat Class II malocclusions [69-71]. When maxillary protraction is necessary, palatal miniscrews can serve as a substitute for miniplates; by using TADs, surgical intervention can be avoided in such cases [72,73]. In Class III correction, buccal shelf miniscrews are an effective option that requires less invasive surgery for placement compared to other anchorage methods [74-76]. Almost all intra-arch mechanics can be sufficiently supported by miniscrews positioned in inter-radicular spaces [77]. A summary of the clinical application of TADs is shown in Figure 9.

Types of tooth movements

Controlled Tipping

The placement of short hooks should be positioned below the crest, with the line of force directed accordingly. To achieve extra labial crown torque and root repositioning, miniscrews may be positioned in the anterior region to provide the required torque for intrusion and root alignment [20,78].

Translation

In orthodontics, when using low-pull mechanics, a posterior open bite may develop. This can be addressed by applying box elastics to the posterior region. Additionally, some experts recommend using anterior bite planes to prevent the deepening of the anterior teeth. In high-pull mechanics, if the line of force passes above the center of resistance (CRes) of the dentition, it can cause a mesial drag on the molars without applying retraction force to the molar hook. To prevent this issue, a transpalatal arch (TPA) is frequently utilized. Furthermore, there is a possibility that an anterior open bite may develop, which can be addressed through the use of anterior box elastics. The symmetrical placement of temporary anchorage devices (TADs) during anterior retraction is an important biomechanical factor. If the implant is placed asymmetrically, the force vectors may cause a tilt of the occlusal plane. This situation should be prevented. Using a cantilever intrusion spring on the side of the lower implant can be a helpful method to prevent occlusal canting [20,78].

Molar Protraction

If the force is applied away from the center of resistance (CRes) of the molar, it will lead to rotation in all three dimensions. Mesial tipping can occur when a force is exerted through a labial hook. The placement of mini-implants at the apex can lead to posterior teeth intrusion, especially if the force vector has an intrusive element. The cant of the occlusal plane may result from unilateral intrusion, requiring control over both vertical and tipping forces. To reduce mesial tilting and intrusion, an SS wire can be used to maintain control. However, this may not always be effective for long-term protraction. The insertion of a lever arm into the auxiliary tube of the first molar aids in countering these forces [20,78].

Distalization of Molar

The tuberosity and the most posterior cortical bones of both the maxilla and mandible are limits for molar distalization. The extraction of third molars might be required, based on the amount of space and the potential for impaction. The distal force must be applied in three dimensions via the point of resistance of the molar for the initiation of movement. The second molars should be the primary targets for distalization since they are easier to manage in all three dimensions. The second molar's root surfaces are large and difficult to control, which can lead to rotation if force is not directed properly. Open coil springs are used to apply distal force to the molar, activated by a ligature attached to the temporary skeletal anchorage device (TSAD). Once molars are distalized, anterior and premolar retraction can be initiated using closed coil springs connected to the same TSAD. Distal vertical elastics help control any tipping of the molar during distalization. Over time, the TSAD may need to be repositioned as the second premolar roots get close to the screw [20,78].

Mesial Rotation

Since the protraction force is applied from the buccal side, there is a tendency for the molars to rotate mesially. To counteract this, the archwire should be contoured inward starting from the premolar region. Mesially angulated molars are a common issue in adult orthodontics. They can obstruct space for fixed bridges or implants, requiring uprighting to improve space for prosthetics [20,78].

Complications

During insertion, the placement of the miniscrew between the roots may harm the roots of the tooth and the periodontal ligament [79]. If the miniscrew does not adequately penetrate the cortical bone, it may shift beneath the mucosal tissue and along the periosteum. The risk of nerve injury is heightened when inserting miniscrews in specific locations, including the mandibular buccal surface, the maxillary palatal area, and the retromolar area [80,81]. Furthermore, air subcutaneous emphysema may develop if air becomes trapped beneath the skin in regions with loose alveolar tissue, especially within the zygomatic area, the posterior buccal region of the mandible, and the retromolar region. The placement of miniscrews close to the sinuses, such as in the maxillary incisal or posterior dentoalveolar regions, can result in sinus perforation, potentially harming the nasal or maxillary sinuses. Another issue to consider is miniscrew instability; applying excessive torsional forces during insertion can lead to bending, breakage, or microfractures in both the miniscrew and the adjacent bone, thus jeopardizing its stability. Complications can occur during orthodontic loading, such as anchorage failure that results from low bone density or the inadequate thickness of the cortical bone, which can impede the necessary support for maintaining appropriate forces throughout the treatment. There is also a risk of miniscrew migration, particularly when substantial orthodontic forces are applied if the anchorage is not completely secured. Soft tissue issues may arise, including the development of painful sores around the miniscrew, known as aphthous ulceration, as well as the overgrowth of soft tissue that could potentially obscure the miniscrew or its attachments, causing irritation and discomfort. Additionally, inflammation or infection in the soft tissues can lead to peri-implantitis. Complications that may arise during the extraction of the miniscrew include the possibility of the screw fracturing, which can make removal more challenging, and partial osseointegration, where a miniscrew that has somewhat fused with the bone may lead to difficulties and potential harm to the surrounding bone when being taken out. Careful planning and proper technique are crucial for minimizing these risks [82,83].

Recent advances

Recent advances in temporary anchorage devices (TADs) include bicortical microimplant and resorbable screw for orthodontic anchorage. A new bicortical microimplant with two anchorage units for applying forces bilaterally has been developed. The mesial displacement of the posterior teeth in beagle dogs, without rotation, was successfully accomplished using bilateral orthodontic forces. A resorbable implant would function as the anchorage unit and could be easily removed or naturally resorbed by the tissues. Absorbable screws are composed of a resorbable copolymer, which is a polyester derivative of L-lactic and glycolic acids. The poly-L-lactic acid/polyglycolic acid (PLA/PGA) copolymer degrades and resorbs in vivo through hydrolysis into L-lactic and glycolic acids, which are subsequently metabolized by the body. This material is nontoxic, nonirritating, and 100% amorphous, breaking down into carbon dioxide and water during metabolism. The potential advantages of bioresorbable implants include less stress shielding of the bone [84].

## Conclusions

In conclusion, temporary anchorage devices (TADs) have become an indispensable tool in modern orthodontics, significantly enhancing the precision and efficiency of tooth movement. They expand the range of treatable cases, allowing for the correction of complex malocclusions that might be challenging or impossible with traditional methods alone. Although factors such as bone quality, insertion technique, and patient hygiene influence their effectiveness, the advantages of temporary anchorage devices (TADs), such as enhanced control, decreased dependence on the patient, and the possibility of more efficient and comfortable treatment, make them a significant tool in achieving the best orthodontic outcomes and creating beautiful, healthy smiles.
